# Mendelian randomization of gut microbiota and exploratory testing of preventive effect of commercial *Rothia mucilaginosa* in constipated mice

**DOI:** 10.3389/fmicb.2026.1941480

**Published:** 2026-10-12

**Authors:** Peishi Jiang, Xiaoqing Yang, Chenghao Zhang, Yuwei Li, Chen Xu

**Affiliations:** 1 Department of Colorectal Surgery, Tianjin Union Medical Center, The First Affiliated Hospital of Nankai University, Nankai University, Tianjin, China; 2 School of Medicine, Nankai University, Tianjin, China; 3 Tianjin Institute of Urology, The 2nd Hospital of Tianjin Medical University, Tianjin Medical University, Tianjin, China; 4 The Institute of Translational Medicine, Tianjin Union Medical Center of Nankai University, Tianjin, China; 5 Tianjin Institute of Coloproctology, Tianjin, China; 6 Graduate School, Tianjin University of Traditional Chinese Medicine, Tianjin, China

**Keywords:** constipation, gut microbiota, intestinal motility, Mendelian randomization, metabolomics, *Rothia mucilaginosa*

## Abstract

Constipation is linked to gut microbiota dysbiosis, but causal microbial contributors and tractable bacterial interventions remain incompletely defined. This study first performed two-sample Mendelian randomization using Dutch Microbiome Project gut-microbiome Genome-Wide Association Study (GWAS) data (*N* = 7,738) and a European constipation GWAS (*N* = 411,623) to identify gut taxa linked to constipation. Then, *in vitro* metabolomics and a loperamide-induced constipation mouse model were used in male C57BL/6 mice to exploratorily test whether the commercial *Rothia mucilaginosa* strain BNCC363031 prevents loperamide-induced constipation. In an exploratory two-sample MR screen across 207 gut taxa, genetically predicted *Rothia* was nominally associated with lower constipation risk [odds ratio (*OR*) = 0.916, *p* = 0.0041], and the species-level *Rothia mucilaginosa* showed a concordant nominal protective association (*OR* = 0.921, *p* = 0.0232). In mice (*n* = 8 per group), preventive gavage partially restored loperamide-suppressed defecation: the cross-day mean fecal pellet count in the last 3 h per day was higher in the *Rothia mucilaginosa* group (RG) than in model controls (MCs) (7.94 vs. 4.41; *p* = 0.0306) but lower than in normal controls (NCs) (13.16; *p* = 0.00162), whereas body weight; small-intestinal transit; and immunohistochemical zonula occludens-1 (ZO-1), Claudin-1, and mucin 2 (MUC2) H-scores were unchanged. A fecal 16S V4 signal corresponding to *R. mucilaginosa* was detected at a very low abundance in only 2/8 RG mice; *in vitro* metabolomics identified candidates of secreted metabolites, including acetic acid. This study provides a reference for research on *Rothia mucilaginosa* in the context of constipation.

## 1 Introduction

Chronic constipation affects approximately 15% of the global population, yet a substantial proportion of patients respond inadequately to first-line dietary fiber and laxative therapies and require further clinical evaluation (Sharma and Rao, 2017; Bharucha and Lacy, 2020). The high prevalence, chronicity, and treatment-refractory subset together make constipation a persistent burden on healthcare utilization and quality of life.

Accumulating evidence links gut microbiota dysbiosis to constipation, and microbial metabolites—particularly short-chain fatty acids—regulate enteric neurons and gastrointestinal motility (Soret et al., 2010; Pan et al., 2022). This has motivated the development of probiotic interventions, although clinical evidence remains mixed: *Lactobacillus plantarum* Lp3a improved functional constipation in a randomized placebo-controlled trial and a loperamide-induced mouse model (Zhang et al., 2022), whereas a large placebo-controlled trial in adults with functional constipation found no superiority of daily *Bifidobacterium lactis* HN019 over placebo for changes in complete spontaneous bowel movements (Cheng et al., 2024). Broader profiling has reported compositional shifts in constipated patients, including altered abundance of butyrate-producing taxa (Zhuang et al., 2019; Tian et al., 2020).

Existing probiotic trials show strain-specific benefits, yet candidate selection typically relies on mechanistic hypotheses or commercial availability rather than genetic evidence for causal protection. Cross-sectional profiling has identified taxa–constipation associations (Parthasarathy et al., 2016), but observational co-occurrence cannot distinguish cause from effect. Using human genetic data to prioritize candidate protective taxa, followed by exploratory experimental testing with a commercial strain, could complement existing probiotic research with a straightforward candidate selection framework.

Mendelian randomization (MR) uses germline genetic variants as instruments to estimate causal effects that are robust to environmental confounding and reverse causation (Hemani et al., 2018). By combining a gut microbiome genome-wide association study (GWAS)(Lopera-Maya et al., 2022) with a constipation GWAS (Sakaue et al., 2021), two-sample MR can nominate candidate protective taxa for downstream validation. In this study, this framework suggested genetically predicted protective associations of *Rothia* and *Rothia mucilaginosa*—a commensal that produces bioactive secondary metabolites (Uranga et al., 2020)—with constipation. Then the *in vitro* secreted metabolome of the commercial *Rothia mucilaginosa* strain BNCC363031 was characterized and tested preventive intragastric gavage of this strain in a loperamide-induced constipation mouse model, integrating human genetic evidence, metabolomic profiling, and preliminary *in vivo* data.

## 2 Materials and methods

### 2.1 GWAS sources and instruments

This study performed two-sample MR of gut microbiota on constipation in R 4.3.3 using TwoSampleMR 0.7.4 (Hemani et al., 2018). Microbial exposures were derived from the Dutch Microbiome Project gut metagenome GWAS (*N* = 7,738 Europeans; 207 taxa from phylum to species levels), and the outcome was the European constipation GWAS GCST90018829 (*N* = 411,623) (Sakaue et al., 2021; Lopera-Maya et al., 2022). For each taxon, candidate instruments were selected at *p* < 1 × 10^−5^, linkage disequilibrium (LD)-clumped to independence (*r*^2^ < 0.001 within a 10,000 kb window), retained at *F* > 10, harmonized with the outcome data, and screened to remove outcome-associated single-nucleotide polymorphisms (SNPs) at *p* < 1 × 10^−5^ (Burgess and Thompson, 2011; Sanna et al., 2019). This workflow yielded 1,835 harmonized SNPs across 207 taxa for downstream MR estimation.

### 2.2 MR estimation and visualization

For each taxon, causal estimates were generated with up to five MR methods: inverse-variance weighted (IVW), MR-Egger, weighted median, simple mode, and weighted mode. IVW was prespecified as the primary estimator, and taxa meeting the nominal screening threshold of *p* < 0.05 were carried forward to the overview forest plot. The same MR workflow generated Cochran heterogeneity statistics, MR-Egger intercept tests for horizontal pleiotropy, and leave-one-out outputs (Bowden et al., 2015; Burgess et al., 2017). Overall forest, taxon-specific forest, funnel, leave-one-out, and scatter plots for *Rothia* and *Rothia mucilaginosa* were generated to visualize the MR causal estimates and the corresponding sensitivity analyses.

### 2.3 OD600 calibration workflow

For the optical density at 600 nm (OD600)-colony-forming unit (CFU)/mL calibration, the standard strain *Rothia mucilaginosa* BNCC363031 (BeNa Culture Collection, Zhengzhou, China) was processed with a two-axis serial-dilution design in which the G1–G8 vertical chain used two-fold steps and each G stock initiated a D1–D6 10-fold dilution chain (Ben-David and Davidson, 2014; Beal et al., 2020). Selected *D* dilutions were plated at 100 μL per plate and colony counts were read at 48 h. The net OD600 was calculated by subtracting the phosphate-buffered saline (PBS) blank, and calibration points were retained only when the plate count was ≥30 and the raw OD600 was ≥0.06. The retained net OD600 values were regressed against log10(CFU/mL) to derive the laboratory OD600–CFU/mL calibration fit from the two-axis dilution workflow and representative counted plates.

### 2.4 Gavage concentration monitoring

During the preventive phase, RG gavage broth was prepared by inoculating Erlenmeyer flasks containing Columbia liquid medium (BeNa Culture Collection, Zhengzhou, China) 1 day earlier and incubating overnight in a temperature-controlled shaking incubator (ZQPW-70, Labotery Instrument Co., Ltd., Tianjin, China; 37 °C, 120 rpm). The overnight culture was centrifuged at 3,000 rpm for 10 min at 4 °C (GTR16-2, Beijing New Era Beili Medical Devices Co., Ltd., Beijing, China); the supernatant was carefully discarded without disturbing the bacterial pellet, the pellet was resuspended in sterile PBS and thoroughly pipetted to fully disperse the bacteria, then centrifuged again at 3,000 rpm for 10 min for one wash, and resuspended in PBS to yield the gavage suspension. The suspension was prepared in 1-day or 2-day batches (for 2-day batches, the suspension was split into two aliquots and the aliquot for next-day use was stored at 4 °C); on each batch harvest day, an aliquot of the freshly resuspended suspension was diluted 100-fold and measured for OD600 on a microplate reader (Synergy HT, BioTek Instruments, Inc., Winooski, VT, USA), with PBS-blank subtraction to yield the net OD600. Daily source-broth concentrations were back-calculated from the laboratory OD-CFU equation and compared with the predefined 1 × 10^10^ CFU/mL gavage target.

### 2.5 *In vitro* metabolomic profiling

For *in vitro* metabolomic profiling, the *Rothia mucilaginosa* strain BNCC363031 was cultured in Columbia liquid medium at 37 °C, 120 rpm for 18 h in five independent culture tubes together with three independent uninoculated medium tubes in the same shaking incubator; after centrifugation, bacterial supernatant (*n* = 5), bacterial pellet (*n* = 5), and blank medium (*n* = 3) were used as analytical matrices. Raw liquid chromatography–mass spectrometry (LC–MS) data were converted to mzXML with ProteoWizard, and XCMS was used for the peak extraction, peak quantification, and cross-sample peak alignment based on the retention time and mass-to-charge ratio. Untargeted positive- and negative-mode LC–MS data were then analyzed for bacterial supernatant versus uninoculated medium and bacterial pellet versus uninoculated medium comparisons, with log2FC defined as log2(bacterial supernatant/uninoculated medium) or log2(bacterial pellet/uninoculated medium); differential metabolites were called across the full 5,038-metabolite table using |log2FC| ≥ 1 and *p* < 0.05. Targeted LC–MS/MS assays for tryptophan metabolites and short-chain fatty acids were analyzed for bacterial supernatant versus uninoculated medium, with targeted differential metabolites called at |log2FC| ≥ 0.58 and *p* < 0.05. Metabolite identification, quantification, and statistical tabulation were completed in Python 3.5.0 and R 3.4.3, and metabolites that increased in the bacterial pellet and supernatant were intersected by compound name to define dually upregulated candidates from the untargeted and targeted metabolomic analyses.

### 2.6 Preventive intervention design

Six-week-old male C57BL/6 mice (20 ± 2 g, Beijing HFK Bioscience Co., Ltd., Beijing, China) were randomized into three groups—NC (normal control), MC (model control), and RG (*Rothia mucilaginosa* group; *n* = 8 per group)—for a 22-day pure-prevention protocol. The protocol consisted of adaptation on days 0–7, gavage of the *Rothia mucilaginosa* strain BNCC363031 in RG with PBS in NC/MC on days 8–14, loperamide (MedChemExpress, Shanghai, China) administration in MC/RG with CMC-Na vehicle (Aladdin, Shanghai, China) in NC on days 15–20, and sacrifice on day 21. The loperamide schedule used 15 mg/kg on days 15–17; on day 15, capturing the group difference window already required continuing collection until 22:00, and on day 17 the recovery time of fecal output shifted further posterior (possibly related to drug accumulation), and the collection activity may disrupt the circadian rhythm of the mice; the dose was therefore exploratorily reduced to 10 mg/kg on days 18–19 as a refinement-based adjustment to keep the fecal output recovery observation window within the regular light phase; as day 19 observations indicated that the 10 mg/kg dose did not maintain the constipation modeling effect, the dose was restored to 15 mg/kg on day 20 to re-establish the model, with the hourly collection advanced to 09:00 on day 20 (versus 14:00 on days 15, 17, and 19) to extend the fecal output recovery observation window within the regular light phase; on each fecal-collection day, loperamide was administered by gavage before the first hourly collection. All animal experiments were conducted in accordance with the guidelines of the Tianjin Medical Experimental Animal Care, and the protocols were approved by the Institutional Animal Care and Use Committee of Yi Shengyuan Gene Technology (Tianjin) Co., Ltd. (approval number YSY-DWLL-2026033; approval date: April 24, 2026). The 3R principles were followed during this experiment: the mice were housed individually under controlled conditions (20–26 °C, 40–70% humidity, and a 12 h light/12 h dark cycle, except on days 15 and 17, when fecal collection began during the day and continued into the night), were allowed free access to food and water except during the 12 h overnight fasting period beginning at 21:00 on day 20, received non-surgical oral gavage without routine analgesic administration and underwent scheduled fecal collection, and were euthanized using cervical dislocation following isoflurane (RWD Life Science Co., Ltd., Shenzhen, China) induction in an induction chamber (1.5–2% isoflurane in oxygen at 1.5 L/min) on a small-animal anesthesia system (Beijing Zhongshi Dichuang Technology Development Co., Ltd., Beijing, China).

### 2.7 Defecation phenotyping and statistics during adaptation and bacterial intervention

On the three pre-modeling fecal collection days (days 1/7/14), corresponding to the baseline, end of adaptation, and end of bacterial intervention, respectively, each mouse was placed in an individual collection cage at 14:00 and the hourly fecal pellet count and wet mass were recorded for 3 h. To eliminate pseudo-replication from repeated sampling of the same mouse across days, each mouse’s 0–1 h, 1–2 h, and 2–3 h values were averaged across the 3 days within each slot, yielding paired per-mouse slot triplets. These triplets (*n* = 24 mice) entered the subsequent group comparisons.

### 2.8 Body weight and fecal water content

Body weight was recorded on days 1, 7, 14, 15, 17, 19, 20, and 21. Fecal water content was computed on the seven fecal collection days (days 1, 7, 14, 15, 17, 19, and 20) as (total wet mass—total dry mass)/total wet mass × 100 after pooling each mouse’s feces collected on that day and oven-drying the pooled sample at 60 °C for 8 h. For the fecal water content, when a mouse produced no collectable feces on a given collection day, that mouse’s record for that day was treated as a missing value.

### 2.9 Modeling-day hourly plotting and derivation of two analysis windows

For the modeling-day hourly fecal pellet-count and wet-mass analyses, the same NC, MC, and RG cohort (*n* = 8 per group) was assessed. Hourly pellet counts and wet mass were plotted across the available hourly windows for each modeling day, with the day 17 22:00–23:00 lights-off slot annotated by gray shading on the corresponding hourly plots. Two parallel analysis windows were then derived from the same hourly data. First, for each modeling day, the last three hourly collection windows were summed per mouse: 19:00–22:00 on day 15, 20:00–23:00 on day 17, 17:00–20:00 on day 19 and 15:00–18:00 on day 20. The analysis window of the last 3 h was prespecified based on direct observation on days 15 and 17 before any analysis at the group level: in the early hours after dosing, fecal output was possibly partly attributable to a transient stress response from placing mice into a novel collection cage; in the intervening hours, pellet output was sporadic for the MC and RG groups; in the later hours after dosing, pellet counts of the MC and RG groups increased with time, so the last 3 h were selected and applied to all four modeling days for consistency. Second, a common post-dose 5–8 h observation window was defined on the same hourly data to test whether the fecal-output findings depend on the choice of analysis window; on day 15 this window coincides with the last-three-hour window (19:00–22:00), and day 19 has no such window. These per-mouse daily totals were visualized as box plots with jittered points for pellet count and wet mass.

### 2.10 Mouse-level modeling-phase fecal output summary

Building on the per-mouse daily last-three-hour totals from the hourly pellet-count and wet-mass analyses above, one mouse-level pellet-count value and one mouse-level wet-mass value were obtained by averaging each metric across the four modeling days (days 15, 17, 19, and 20) for each mouse in the same NC, MC, and RG cohort (*n* = 8 per group). These mouse-level values were visualized as box plots with jittered points. To assess the robustness of the analysis-window choice and the influence of the dose-reduction day (day 19), two sensitivity-analysis variants were additionally defined on the same hourly data. First, excluding day 19, the last-three-hour totals of the remaining 3 days (days 15, 17, and 20) were averaged per mouse to yield mouse-level pellet-count and wet-mass values. Second, within the common post-dose 5–8 h observation window, per-mouse cross-day means were computed across days 15, 17, and 20 (day 19 excluded because the collection ended before 8 h post-dose) to yield mouse-level pellet-count and wet-mass values.

### 2.11 Small-intestinal carbon transit

On day 21, after a 12 h overnight fast had begun at 21:00 on day 20, all three groups received 0.2 mL of a 50 g/L activated charcoal suspension via gavage. The suspension was prepared by boiling 10 g gum arabic in 80 mL distilled water until clear, adding 5 g activated charcoal powder, reboiling three times to disperse the charcoal, cooling, and making up to 100 mL with distilled water. The planned sacrifice point was about 30 min after the carbon gavage: isoflurane induction (about 2 min) began about 28 min after the gavage, followed at about 30 min by retro-orbital blood collection and euthanasia. Because NC was sacrificed on the planned approximately 30 min schedule, whereas MC and RG were both delayed to approximately 35 min, the small-intestine length was measured in all three groups, but the carbon migration distance and transit rate were compared only between the interval-matched MC and RG. One MC mouse was excluded from the interval-dependent endpoints because the carbon gavage was unsuccessful, leaving MC *n* = 7 for those comparisons.

### 2.12 Pooled-background 16S enrichment testing

Day 13 fecal microbiota from the prevention cohort (NC, MC, and RG) were analyzed using 16S rRNA V4 amplicon sequencing, and genus- and species-level relative abundance tables were used as the input matrices. The two groups without *R. mucilaginosa* gavages (NC and MC) were pooled into a 16-sample background and compared with the 8 RG samples for each taxon. Relative-abundance values were converted to percentages before screening for differential abundance. Taxa were called RG-enriched when *p* < 0.05 and the RG mean exceeded the pooled background mean. Selected taxa were plotted as boxplots with jittered points and group means across NC, MC, and RG using pandas 2.2.3 (data handling), NumPy 1.26.4 (numerical computation), and matplotlib 3.10.0 (plotting). The 16S rRNA gene amplicon sequencing data were deposited in the NCBI Sequence Read Archive (SRA) under BioProject accession PRJNA1478980.

### 2.13 HE imaging and thickness measurement

Distal colon paraffin sections (4 μm) from NC, MC, and RG mice (*n* = 8 per group) were stained with Harris hematoxylin and eosin (hematoxylin, ZSGB-BIO ZLI-9610; eosin, ZSGB-BIO ZLI-9613) and digitized on a 3DHISTECH P250 FLASH scanner as .mrxs whole-slide images. Whole-slide browsing and measurement annotation were performed in QuPath version 0.6.0 (Bankhead et al., 2017). Muscle-layer thickness was measured manually by drawing four lines across the muscularis on each slide and averaging the four line lengths to obtain one mouse-level value.

### 2.14 IHC sampling and H-score workflow

Distal colon paraffin sections from NC, MC, and RG mice were immunohistochemically stained for ZO-1, Claudin-1, and MUC2, yielding 24 whole-slide scans per marker (3DHISTECH Pannoramic Scan). Staining used rabbit polyclonal primary antibodies (ZO-1, Proteintech 21,773-1-AP, 1:2000; Claudin-1, Proteintech 28,674-1-AP, 1:1000; MUC2, Proteintech 27,675-1-AP, 1:2000), ethylenediamine tetra-based antigen retrieval (pH 9.0), 3,3′-diaminobenzidine (DAB) chromogen, and hematoxylin counterstain (Solarbio G1140). For each IHC-stained slide, three random circular high-power fields were sampled within the stained tissue region (300 μm diameter). The same QuPath version was used for positive cell detection with hematoxylin–3,3′-diaminobenzidine (H-DAB) color deconvolution and classification of detected cells by mean cytoplasmic DAB optical density as negative, 1+, 2+, or 3+. H-scores were computed on a 0–300 scale as 1 × %(1+) + 2 × %(2+) + 3 × %(3+), using intensity cutoffs of 0.05/0.10/0.20 for ZO-1 and Claudin-1 and 0.10/0.20/0.40 for MUC2; the three high power field (HPF)-level scores were then averaged to give one mouse-level H-score.

### 2.15 Statistical analysis

For the *in vivo* experiments, all statistics were performed in Python 3.10.16 with SciPy 1.15.1, pandas 2.2.3, NumPy 1.26.4 and matplotlib 3.10.0, with a significance threshold of *P* < 0.05, and continuous outcomes were summarized as mean ± SEM. Body weight and fecal water content, as daily single values, were compared across NC, MC, and RG by one-way analysis of variance (ANOVA) as the omnibus test, which is robust to non-normality under balanced group sizes. The principal pairwise comparison was prespecified as RG versus MC (testing whether preventive *Rothia mucilaginosa* strain BNCC363031 gavage rescues the loperamide-induced constipation phenotype). Pairwise contrasts between NC, MC, and RG were tested by a Levene-guided rule (two-sided Student’s *t* when Levene *p* ≥ 0.05, otherwise two-sided Welch *t*) to accommodate unequal variances under small samples, with raw *p* values reported without multiple-comparison correction; for the principal comparison (RG vs. MC), Cohen’s *d* and its 95% confidence interval were additionally reported. This rule was applied to the following outcomes: (i) daily-level metrics on each modeling day, including fecal water content, the last-3-h fecal pellet count and wet mass as a per-day total, and the 5–8 h post-dose fecal pellet count and wet mass as a per-day total (day 19 excluded for the 5–8 h metric because collection ended before 8 h post-dose); (ii) per-mouse cross-day means, including the last-3-h daily totals across the four modeling days, the last-3-h daily totals across the three modeling days excluding day 19, and the 5–8 h post-dose daily totals across the three modeling days excluding day 19; and (iii) endpoint metrics, including small-intestinal length across NC, MC, and RG, and small-intestinal transit rate and carbon migration distance between MC and RG. Endpoint (day 21) HE muscle-layer thickness and IHC H-scores (ZO-1, Claudin-1, MUC2) were compared between groups by two-sided Welch *t* tests. Per-mouse slot triplets (0–1 h, 1–2 h, 2–3 h), as hourly-slot values averaged across the three pre-modeling days (*n* = 24), were compared across the three slots by two-sided paired *t*-tests (each mouse providing one paired value per slot). Given the non-normal, zero-inflated nature of microbiome relative abundance data, day-13 16S relative abundance was compared between the pooled background (NC and MC) and RG by two-sided Mann–Whitney *U* tests.

## 3 Results

### 3.1 Two-sample MR suggests a nominally significant protective association of *Rothia* with constipation

Across 207 testable taxa (1,835 harmonized SNPs; constipation outcome *N* = 411,623), IVW estimates were obtained for 203 taxa; the remaining four retained only one instrument after harmonization and thus yielded no IVW estimate. IVW flagged 15 nominally significant associations (*P* < 0.05; nine protective, OR<1; six risk, OR>1) (Figure 1A). These 15 associations were selected by uncorrected IVW *p* < 0.05 without multiple-testing correction across the 203 IVW tests; BH-FDR-adjusted *p* values for the complete IVW screening are provided in Supplementary Table S4. The strongest protective signal was the genus *Rothia* and its family Micrococcaceae, with identical estimates from six shared SNPs [OR = 0.916, 95% confidence interval (CI) 0.863–0.973, *p* = 0.0041], whereas Prevotellaceae (OR = 1.132, *p* = 0.0091) and *Bacteroides plebeius* (OR = 1.069, *p* = 0.0118) led the risk associations. Per-SNP estimates for the genus *Rothia* (six SNPs) and the species *R. mucilaginosa* (four SNPs) were mostly protective (Figures 1B,C); for the genus, five out of six SNPs indicated protection, and the IVW (OR = 0.916, 95% CI 0.863–0.973, *p* = 0.0041) and weighted-median (OR = 0.914, *p* = 0.024) pooled estimates indicated protection in the same direction, while the species IVW pooled estimate also indicated protection (OR = 0.921, 95% CI 0.857–0.989, *p* = 0.0232). A separate protective branch involved the Firmicutes lineage, including the phylum Firmicutes (OR = 0.870, *p* = 0.0190) and the Clostridia class/Clostridiales order (OR = 0.885, *p* = 0.0298). For *Rothia mucilaginosa* and the genus *Rothia*, funnel plots were approximately symmetric (Figures 1D,E), MR-Egger intercept tests were non-significant (*R. mucilaginosa p* = 0.932; genus *Rothia p* = 0.212), leave-one-out analyses showed that neither IVW pooled estimate depended on a single SNP (Figures 1F,G), and scatter plots showed negative IVW slopes for both exposures (*R. mucilaginosa β* = −0.083; genus *Rothia β* = −0.088), whereas the genus MR-Egger slope was positive (*β* = +0.115), with a wide 95% CI (0.853–1.477) (Figures 1H,I). Three taxa (*Pseudoflavonifractor capillosus*, *Pseudoflavonifractor*, and *Dorea longicatena*) showed severe heterogeneity (Q_pval < 0.001) and were excluded from the core conclusions.

**Figure 1:**
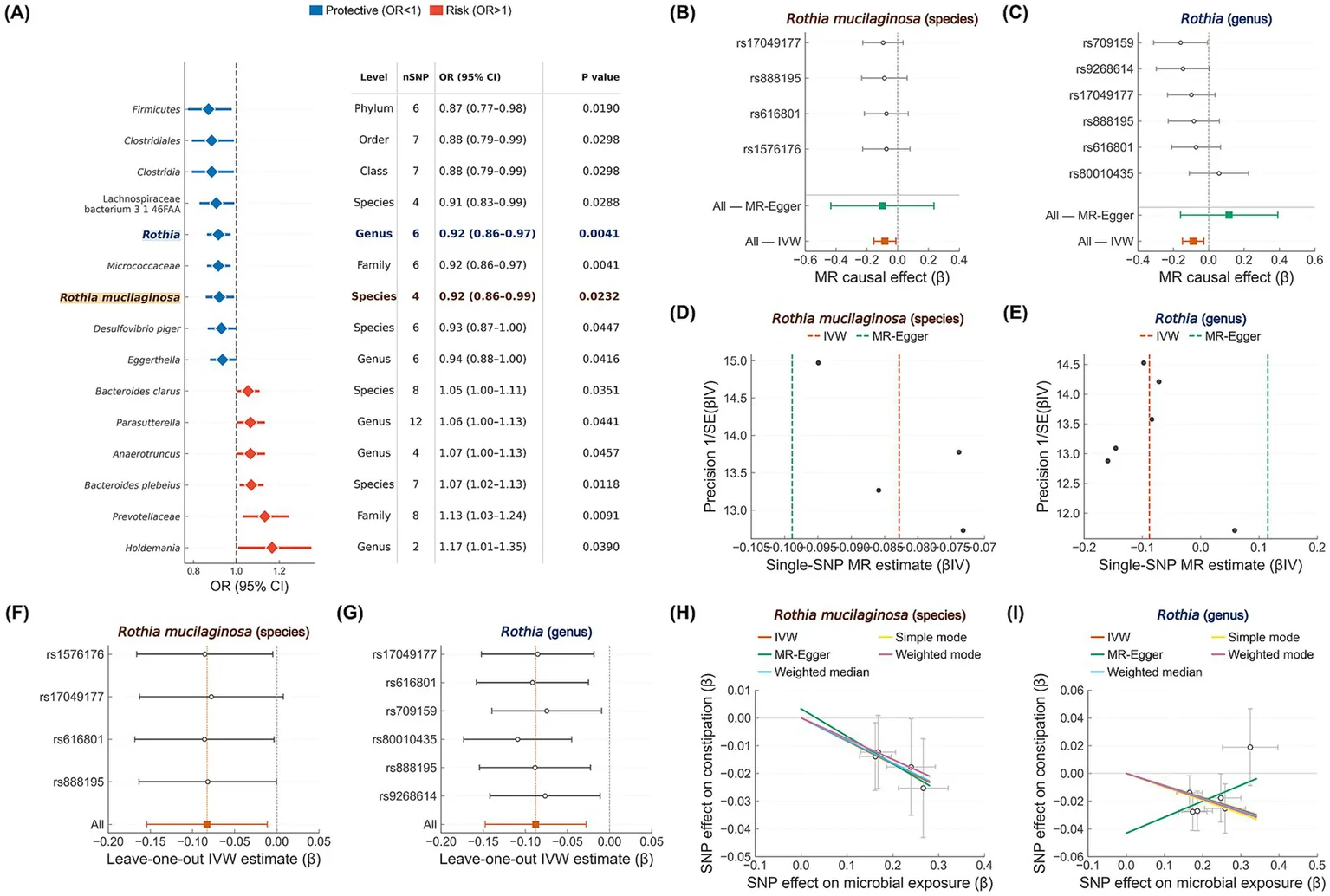
Two-sample MR of gut microbiota on constipation. **(A)** Forest plot of gut microbiota taxa with IVW *p* < 0.05. **(B)** Forest plot for *R. mucilaginosa*. **(C)** Forest plot for *Rothia*. **(D)** Funnel plot for *R. mucilaginosa*. **(E)** Funnel plot for *Rothia*. **(F)** Leave-one-out analysis for *R. mucilaginosa*. **(G)** Leave-one-out analysis for *Rothia*. **(H)** Scatter plot for *R. mucilaginosa*. **(I)** Scatter plot for *Rothia*. MR, Mendelian randomization; IVW, inverse-variance weighted; SNP, single-nucleotide polymorphism; CI, confidence interval; SE, standard error.

### 3.2 OD600 calibration and daily gavage dose verification

The OD600–CFU/mL calibration workflow for *Rothia mucilaginosa* strain BNCC363031 comprised the G1–G8 two-fold gradient and the D1–D6 10-fold dilution chains, with 48 h colony counts from G1–D5, G2–D5, G3–D5, G4–D4, G5–D4, G6–D4, G7–D4, and G8–D3 (Figures 2A,B). After excluding G3–D5 and G7–D4 for 48 h colony counts < 30 and G8–D3 for raw OD600 < 0.06, G1–D5, G2–D5, G4–D4, G5–D4, and G6–D4 (five gradient–dilution points) were retained for the calibration fit. Linear regression yielded log10(CFU/mL) = 2.395 × OD600(net) + 6.730, with *R^2^* = 0.931, *p* = 0.0078, and *n* = 5 (Figure 2C). The daily net OD600 of the gavage broth on days 8–14 ranged from 0.226 to 0.472, which is below the reference OD of approximately 0.530 for the 1 × 10^10^ CFU/mL target at 100 × dilution (Figure 2D). The lowest readings occurred on days 8 and 9 (0.226), and values from day 10 onward stabilized at 0.44–0.47. Back-calculation gave 1.87 × 10^9^ CFU/mL for days 8 and 9 and 6.15 × 10^9^–7.25 × 10^9^ CFU/mL for days 10–14.

**Figure 2:**
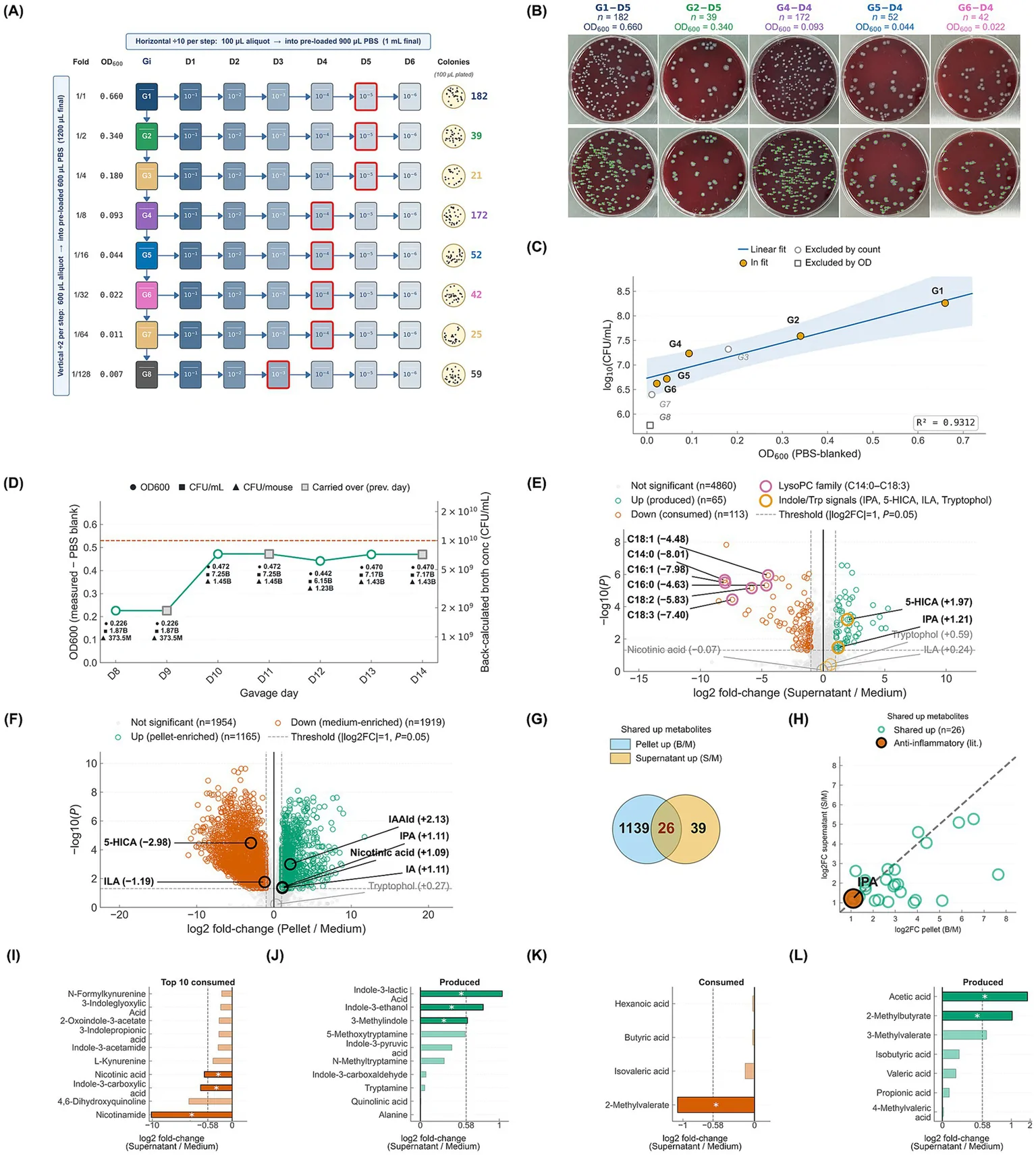
*Rothia mucilaginosa* strain BNCC363031 OD600–CFU calibration, gavage-dose verification, and metabolomic profiling. **(A)** Two-axis serial dilution for OD600–CFU/mL calibration. **(B)** Primary plates for G1, G2, G4, G5, and G6. **(C)** OD600–CFU/mL calibration curve. **(D)** Daily net OD600 of gavaged *R. mucilaginosa* broth on days 8–14. **(E)** Supernatant versus medium volcano plot. **(F)** Pellet versus medium volcano plot. **(G)** Overlap of upregulated metabolites in pellet and supernatant. **(H)** Pellet versus supernatant log2FC scatter for shared upregulated metabolites. **(I)** Targeted tryptophan panel supernatant versus medium log2FC bar plot of decreased metabolites. **(J)** Targeted tryptophan panel supernatant versus medium log2FC bar plot of increased metabolites. **(K)** Targeted short-chain fatty acid supernatant versus medium log2FC bar plot of decreased metabolites. **(L)** Targeted short-chain fatty acid supernatant versus medium log2FC bar plot of increased metabolites. *Indicates *p* < 0.05 in (**I**–**L**). OD600, optical density at 600 nm; CFU, colony-forming unit; PBS, phosphate-buffered saline; FC, fold change; S, supernatant; M, medium; B, pellet; LysoPC, lysophosphatidylcholine; IPA, indole-3-propionic acid; ILA, indole-3-lactic acid; 5-HICA, 5-hydroxyindole-2-carboxylic acid; IAAld, indole-3-acetaldehyde; IA, indoleacrylic acid.

### 3.3 Untargeted and targeted metabolomic output

The supernatant versus medium secretome contained 178 differential metabolites, including 65 net-produced and 113 net-consumed metabolites, with the strongest net consumption signals concentrated in the lysophosphatidylcholine (LysoPC) chain C14:0-C18:3 (Figure 2E). The pellet versus medium profile was broader, with 3,084 differential metabolites, including 1,165 up and 1,919 down (Figure 2F). Intersecting the pellet-up and supernatant-up sets yielded 26 shared upregulated metabolites, representing 40.0% of the 65 supernatant-up metabolites (Figure 2G). Among the shared upregulated metabolites was indole-3-propionic acid (IPA), (Figure 2H); however, in the targeted tryptophan panel comparing supernatant to medium, IPA (listed as 3-indolepropionic acid) did not reach the differential threshold and showed the opposite direction to the untargeted result. Targeted tryptophan LC–MS/MS detected 34 metabolites and identified 6 significant changes: Indole-3-lactic acid (ILA), Indole-3-ethanol, and 3-methylindole were net-produced, whereas nicotinamide, indole-3-carboxylic acid, and nicotinic acid were net-consumed (Figures 2I,J). Matching these six to the untargeted supernatant and pellet datasets by standard database identifiers (HMDB/KEGG/CAS) located three: ILA (as indolelactic acid; non-significant in the supernatant, significantly decreased in the pellet), tryptophol (as indole-3-ethanol; non-significant in both), and nicotinic acid (as niacin acid VB3; non-significant in the supernatant, significantly increased in the pellet), while 3-methylindole, indole-3-carboxylic acid and nicotinamide had no untargeted match (Figures 2E,F). Targeted short-chain fatty acid LC–MS/MS detected 11 metabolites and identified three significant changes: acetic acid and 2-methylbutyrate were net produced, whereas 2-methylvalerate was net consumed (Figures 2K,L).

### 3.4 Novel environment defecation burst

In this pure prevention protocol, RG received *Rothia mucilaginosa* strain BNCC363031 by gavage before loperamide modeling while NC received a vehicle, and on the three pre-modeling days (days 1/7/14), feces were collected hourly for 3 h after each mouse entered an individual cage (Figure 3A). Pooling all mice across these days, the first-hour fecal output far exceeded the two later slots for pellet count and wet mass (Figures 3B–I). The pellet count averaged 4.50 ± 0.30 in the first hour versus 1.93 ± 0.21 and 1.56 ± 0.23 in hours 2 and 3, with the first hour contributing ~56% of the 3 h total; paired *t*-tests showed higher first-hour output (H1 vs. H2: *p* < 0.001; H1 vs. H3: *p* < 0.001; Figure 3H). Wet mass showed the same pattern (118.36 ± 7.74 vs. 41.10 ± 4.55 and 26.89 ± 4.04 mg; ~64% of the 3 h total); paired *t*-tests showed higher first-hour output (H1 vs. H2: *p* < 0.001; H1 vs. H3: *p* < 0.001; Figure 3I), and the daily mouse-level source data are provided in Supplementary Table S1.

**Figure 3:**
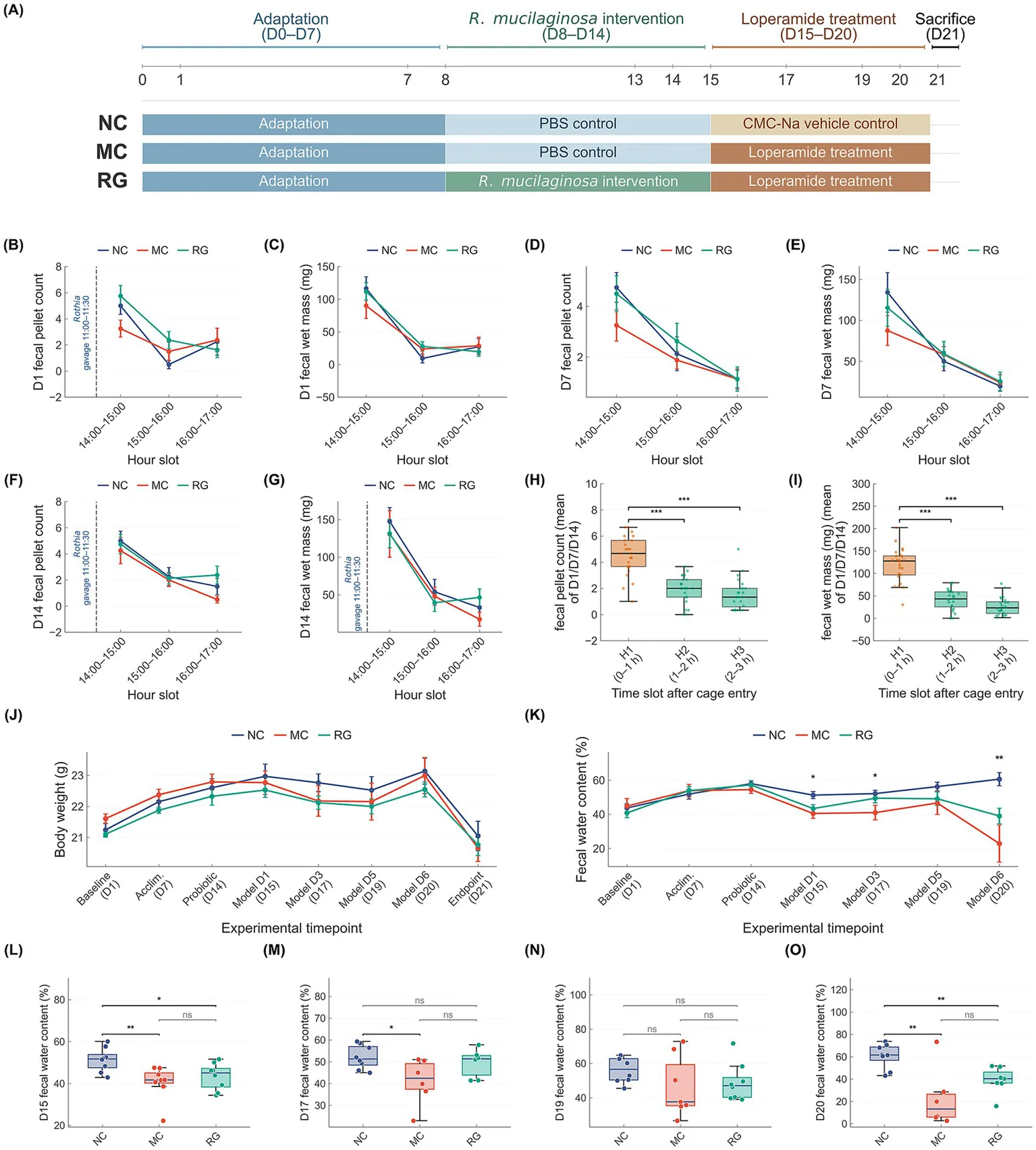
Preventive *Rothia mucilaginosa* strain BNCC363031 intervention and effects on defecation and fecal water content in NC, MC, and RG mice. **(A)** Experimental timeline. **(B)** Hourly fecal pellet count on day 1. **(C)** Hourly fecal wet mass on day 1. **(D)** Hourly fecal pellet count on day 7. **(E)** Hourly fecal wet mass on day 7. **(F)** Hourly fecal pellet count on day 14. **(G)** Hourly fecal wet mass on day 14. **(H)** Fecal pellet counts across 0–1/1–2/2–3 h slots after cage placement. **(I)** Fecal wet masses across 0–1/1–2/2–3 h slots after cage placement. **(J)** Body weight time course. **(K)** Fecal water content time course. **(L)** Fecal water content on day 15. **(M)** Fecal water content on day 17. **(N)** Fecal water content on day 19. **(O)** Fecal water content on day 20. *p < 0.05, **p < 0.01, ***p < 0.001. NC, normal control; MC, model control; RG, *R. mucilaginosa* group; SEM, standard error of the mean; CMC-Na, sodium carboxymethyl cellulose.

### 3.5 Body weight and fecal water content

Body weight showed no between-group difference at any of the eight timepoints (all one-way ANOVA *p* > 0.05; Figure 3J). Across the collection days, one-way ANOVA showed no significant omnibus differences between NC, MC, and RG on day 1 (*p* = 0.6842), day 7 (*p* = 0.861), or day 14 (*p* = 0.5135), whereas during the loperamide modeling phase, declining fecal water content in MC and RG relative to NC was evident during the time course (Figure 3K). One-way ANOVA showed significant omnibus differences on day 15 (*p* = 0.0135), day 17 (*p* = 0.0417), and day 20 (*p* = 0.0024), but not day 19; the MC and RG fecal water contents were lower than that of NC on days 15 and 20, only MC was lower than NC on day 17, and no pairwise difference reached significance on day 19 (loperamide dose was 15 mg/kg on days 15, 17, and 20, and 10 mg/kg on day 19) (Figures 3L–O). For the principal comparison RG vs. MC on fecal water content, RG was numerically higher than MC on all four modeling days, yet pairwise *t* tests reached no significance on any day (day 15: *p* = 0.4423, Cohen’s *d* = 0.395, 95% CI -0.687–1.478; day 17: *p* = 0.1275, *d* = 0.960, 95% CI -0.399–2.318; day 19: *p* = 0.7475, *d* = 0.170, 95% CI -0.950–1.290; day 20: *p* = 0.1751, *d* = 0.806, 95% CI -0.467–2.079).

### 3.6 Hourly fecal output and two analysis window totals during modeling

On days 15 and 17 (loperamide 15 mg/kg), MC and RG produced consistently fewer hourly pellets than NC (Figures 4A,C); this suppression attenuated on day 19, when the dose was lowered to 10 mg/kg, with both model groups transiently exceeding NC in late-afternoon slots (Figure 4E), and the suppression largely resumed on day 20 after restoration to 15 mg/kg, with MC and RG again falling below NC across most slots (Figure 4G). Per-mouse last-3-h pellet totals showed NC > MC and NC > RG on days 15 and 17 (all *p* < 0.05), with RG > MC only on day 15 (*p* = 0.0491, Cohen’s *d* = 1.078, 95% CI -0.070–2.225); no pairwise contrast reached significance on days 19 or 20, and RG remained numerically higher than MC on days 17, 19, and 20 without reaching significance (Figure 4B,D,F,H). Under the common 5–8 h post-dose window, RG versus MC on pellet count was numerically higher on day 17 but lower on day 20, neither reaching significance (Figures 5E,G). On days 15 and 17 (15 mg/kg), the hourly fecal wet-mass curves largely tracked the pellet-count trajectories, with MC and RG mostly below NC; on day 19 (10 mg/kg) and day 20 (15 mg/kg), the wet-mass curves remained largely consistent with the pellet-count trajectories (Figure 6A,C,E,G). Per-mouse last-3-h wet-mass totals showed NC > MC and NC > RG on days 15 and 17 (all *p* < 0.05), with RG > MC only on day 15 (*p* = 0.0291, Cohen’s *d* = 1.215, 95% CI 0.048–2.382); on day 20 only NC > MC reached significance (*p* = 0.0449), and RG remained numerically higher than MC on days 17, 19, and 20 without reaching significance (Figure 6B,D,F,H). Under the common 5–8 h post-dose window, RG versus MC on wet mass was numerically higher on day 17 but lower on day 20, neither reaching significance (Figures 5F,H).

**Figure 4:**
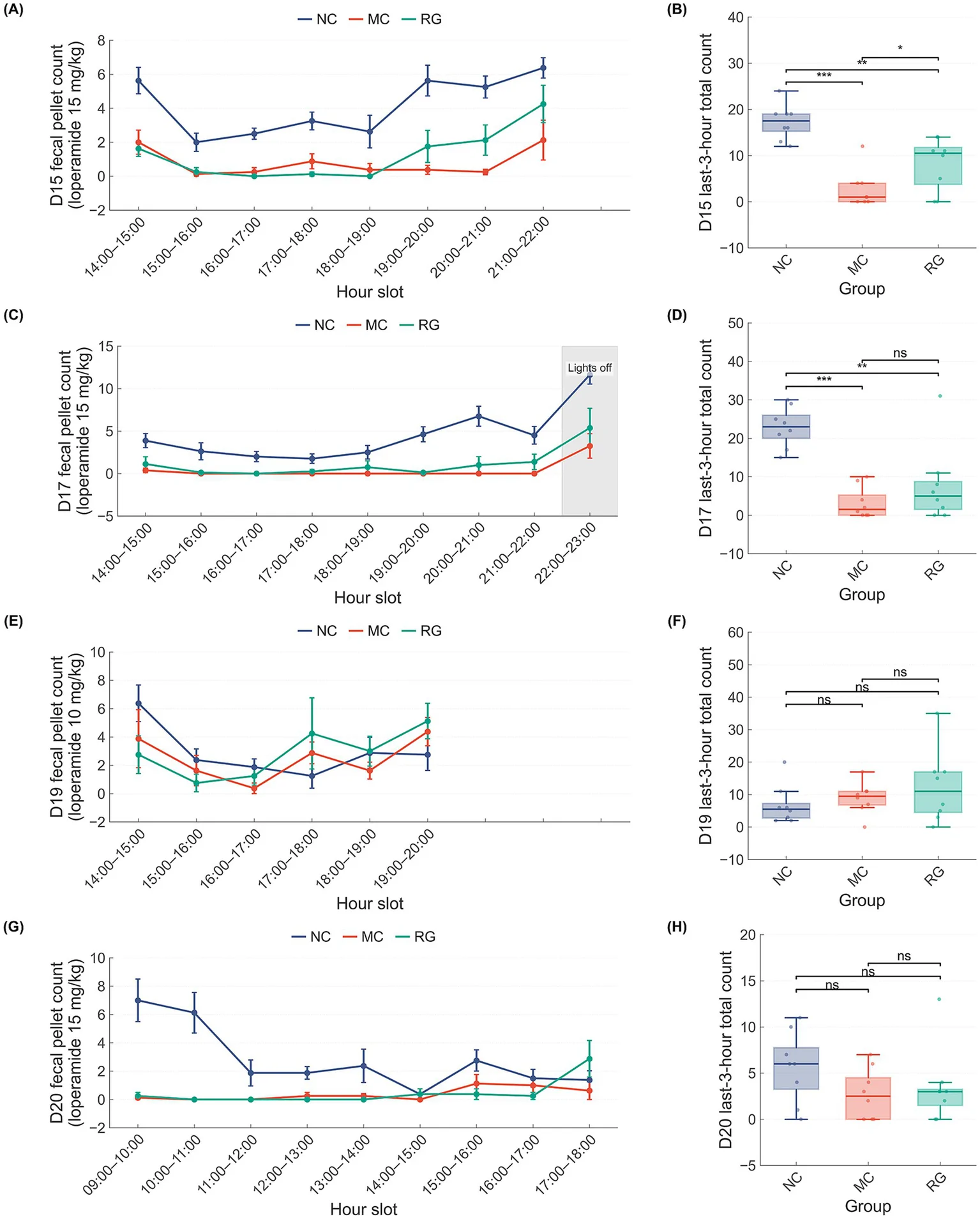
Hourly fecal pellet output across four loperamide modeling days in NC, MC, and RG mice. **(A)** Hourly fecal pellet count on day 15. **(B)** Last-3-h fecal pellet count on day 15. **(C)** Hourly fecal pellet count on day 17. **(D)** Last-3-h fecal pellet count on day 17. **(E)** Hourly fecal pellet count on day 19. **(F)** Last-3-h fecal pellet count on day 19. **(G)** Hourly fecal pellet count on day 20. **(H)** Last-3-h fecal pellet count on day 20. *p < 0.05, **p < 0.01, ***p < 0.001. NC, normal control; MC, model control; RG, *R. mucilaginosa* group; SEM, standard error of the mean; PBS, phosphate-buffered saline.

**Figure 5:**
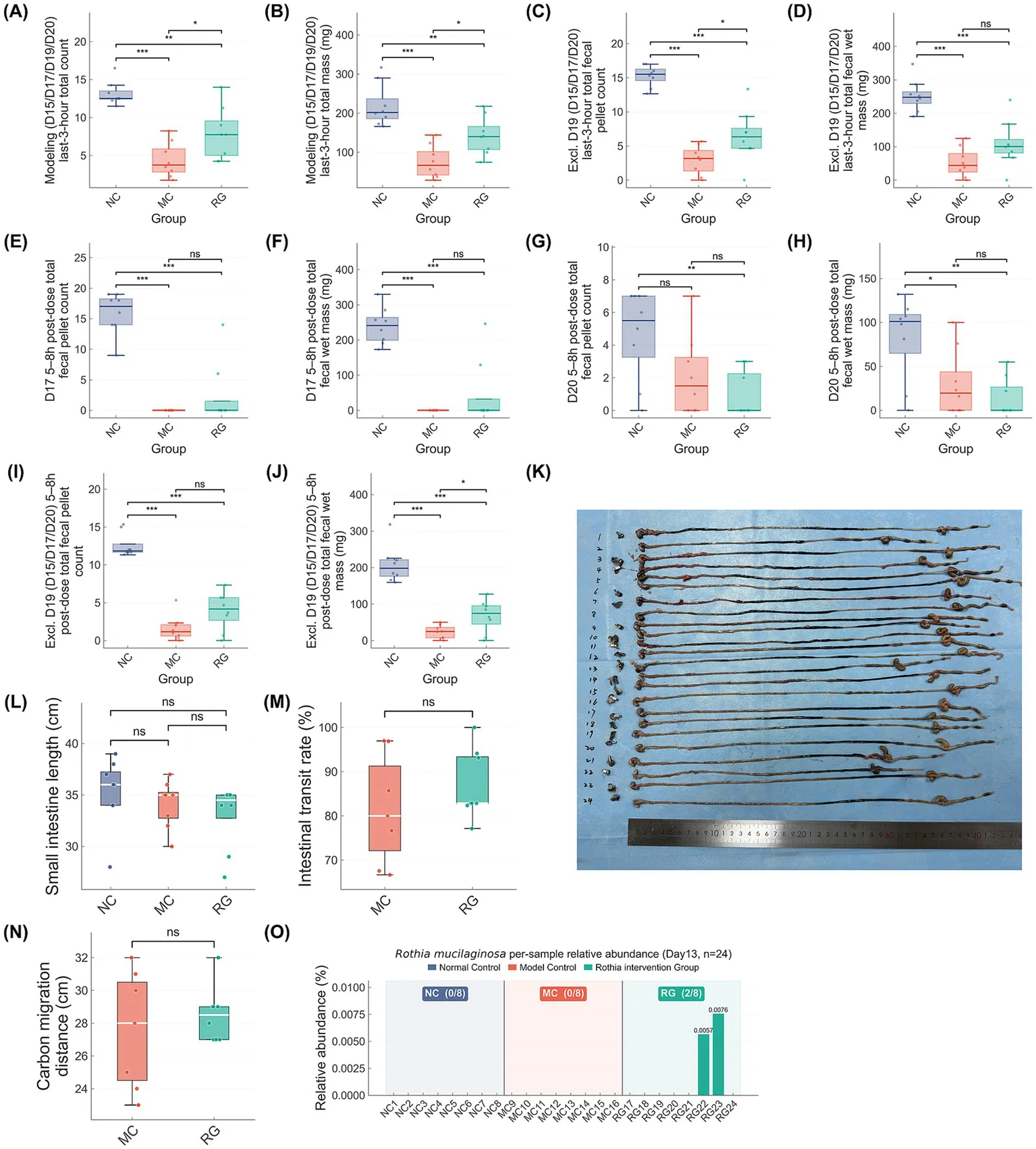
Fecal output (main and sensitivity analyses), small-intestinal transit, and day 13 fecal 16S V4 signal corresponding to *Rothia mucilaginosa* strain BNCC363031 in NC, MC, and RG mice (*n* = 8 per group). **(A,B)** Last-3-h fecal pellet count and wet mass averaged across modeling days (D15/D17/D19/D20) per mouse. **(C,D)** Sensitivity analysis excluding the reduced-dose day 19: last-3-h pellet count and wet mass (per-mouse mean across D15/D17/D20). **(E,F)** Fixed 5–8 h post-dose window fecal pellet count and wet mass on day 17. **(G,H)** Fixed 5–8 h post-dose window fecal pellet count and wet mass on day 20. **(I,J)** Sensitivity analysis: fixed 5–8 h post-dose window excluding day 19, pellet count and wet mass (per-mouse mean across D15/D17/D20). **(K)** Gross photograph of the small-intestinal carbon-transit assay. **(L)** Small-intestine length. **(M,N)** Small-intestinal transit rate and carbon migration distance (MC vs. RG). **(O)** Day 13 per-sample fecal 16S V4 signal corresponding to *R. mucilaginosa* (*n* = 24). *p < 0.05, **p < 0.01, ***p < 0.001. NC, normal control; MC, model control; RG, *R. mucilaginosa* group.

**Figure 6:**
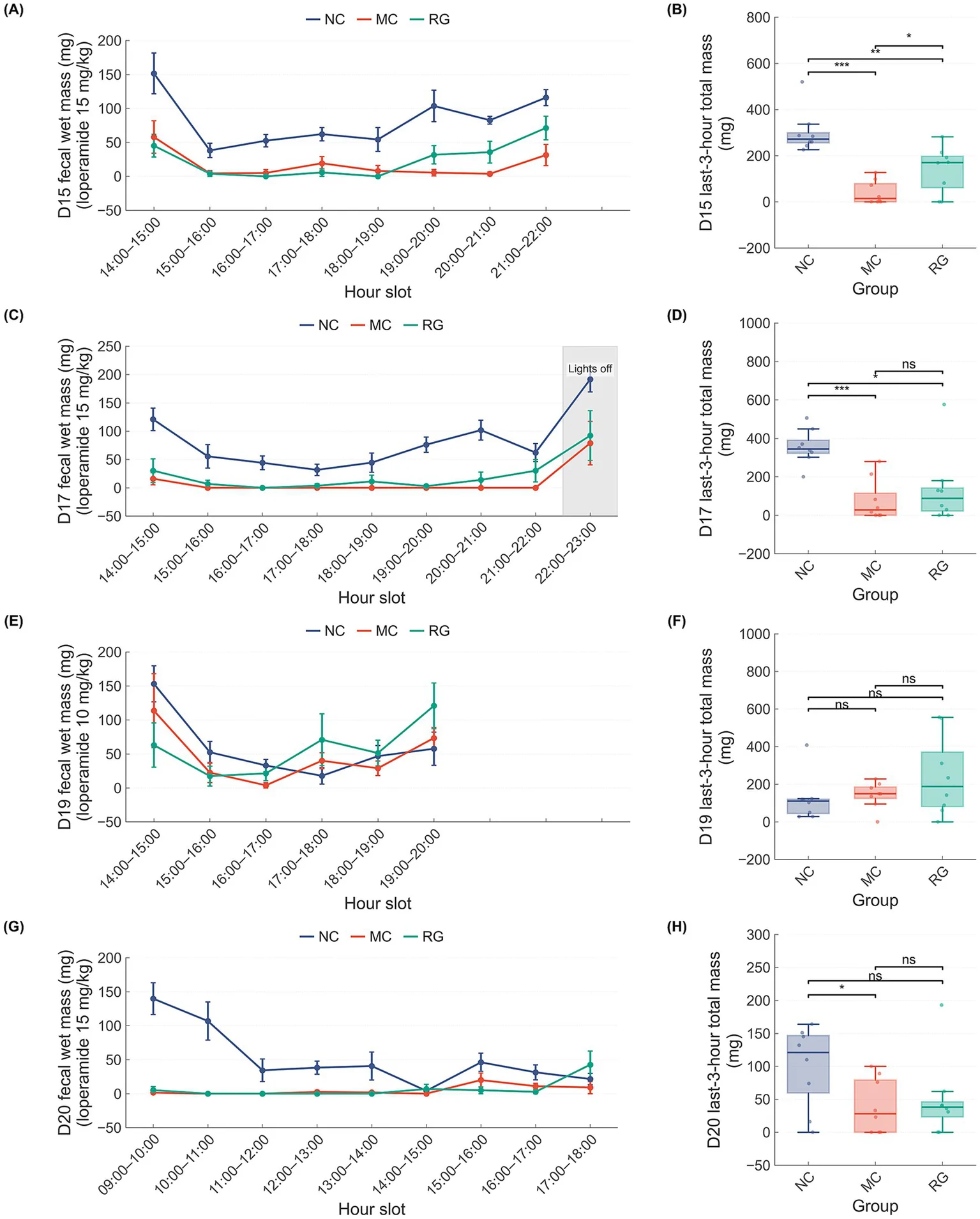
Hourly fecal wet mass during loperamide modeling in NC, MC, and RG mice. **(A)** Hourly fecal wet mass on day 15. **(B)** Last-3-h fecal wet mass on day 15. **(C)** Hourly fecal wet mass on day 17. **(D)** Last-3-h fecal wet mass on day 17. **(E)** Hourly fecal wet mass on day 19. **(F)** Last-3-h fecal wet mass on day 19. **(G)** Hourly fecal wet mass on day 20. **(H)** Last-3-h fecal wet mass on day 20. *p < 0.05, **p < 0.01, ***p < 0.001. NC, normal control; MC, model control; RG, *R. mucilaginosa* group; SEM, standard error of the mean; PBS, phosphate-buffered saline.

### 3.7 Fecal output during the modeling phase by per mouse means across days and sensitivity to analysis window

Building on the hourly trajectories above, per-mouse cross-day means were computed to summarize the modeling-phase fecal output, and pairwise *t* tests on the per-mouse cross-day mean of the summed last-3-h fecal pellet count across days 15/17/19/20 showed that RG (7.94) exceeded MC [4.41; *t* = −2.41, *p* = 0.0306, Cohen’s *d* = 1.203, 95% CI (0.037, 2.368)] but remained below NC (13.16; *t* = 3.89, *p* = 0.00162) (Figure 5A). For fecal wet mass, the per-mouse cross-day mean showed that RG (142.03 mg) was higher than MC [75.47 mg; *t* = −2.91, *p* = 0.0114, Cohen’s *d* = 1.455, 95% CI (0.249, 2.660)] but lower than NC (219.69 mg; *t* = 2.99, *p* = 0.00981) (Figure 5B). To assess the robustness of the analysis-window choice and the influence of the dose-reduction day (day 19), two sensitivity variants were examined on the same hourly data. Under the last-3-h window with day 19 excluded (days 15/17/20, per-mouse mean across the 3 days), RG versus MC on pellet count remained significant [*t* = −2.270, *p* = 0.0396, mean difference 3.542, 95% CI (0.195, 6.889), Cohen’s *d* = 1.135, 95% CI (−0.021, 2.290)] (Figure 5C), whereas wet mass did not reach significance [*t* = −1.872, *p* = 0.0822, mean difference 55.208, 95% CI (−8.030, 118.447), Cohen’s *d* = 0.936, 95% CI (−0.193, 2.066)] (Figure 5D). Under the common post-dose 5–8 h window (days 15/17/20), RG versus MC on pellet count did not reach significance [*t* = −2.092, *p* = 0.0551, Cohen’s *d* = 1.046, 95% CI (−0.097, 2.190)] (Figure 5I), while wet mass remained significant [*t* = −2.521, *p* = 0.0245, Cohen’s *d* = 1.260, 95% CI (0.086, 2.435)] (Figure 5J).

### 3.8 Small-intestinal transit

Gross photographs of small-intestinal carbon-transit preparations from all 24 mice are shown (Figure 5K). RG did not alter any small-intestinal measure: length was comparable across the three groups [NC 35.2, MC 34.1, RG 33.0 cm; RG versus MC *t* = 0.814, *p* = 0.4291, Cohen’s *d* = −0.407, 95% CI (−1.491, 0.676)] (Figure 5L), and neither the transit rate [MC 81.5%, RG 86.9%; RG versus MC *t* = −1.023, *p* = 0.3249, Cohen’s *d* = 0.530, 95%CI (−0.608, 1.667)] (Figure 5M) nor carbon migration distance [MC 27.6, RG 28.5 cm; RG versus MC *t* = −0.625, *p* = 0.5488, Cohen’s *d* = 0.339, 95% CI (−0.787, 1.465)] (Figure 5N) differed between MC and RG (all *p* > 0.05).

### 3.9 *Rothia* detection and taxa enriched in RG across genus and species levels

On day 13, the fecal 16S V4 signal corresponding to the *Rothia mucilaginosa* strain BNCC363031 was detected only in RG (2/8 mice; 0/8 in NC and MC) and at very low abundance (RG mean 0.00166%, maximum 0.00759%) (Figure 5O). At the genus level, four taxa met the RG-enrichment criterion (Figures 7A–D): *Segatella* increased from a pooled-background mean of 0.0306 to 1.5071% in RG (*p* = 0.0051; Figure 7A). The Prevotellaceae NK3B31 group was undetected in the pooled background and reached 0.0420% in RG (*p* = 0.0123; Figure 7B). *Bacteroides* increased from 2.9118 to 7.3339% (*p* = 0.0448; Figure 7C), while *Rothia* increased from 0.0000 to 0.0017% (*p* = 0.0478; Figure 7D). At the species level, three RG-enriched taxa are displayed in Figure 7E–G, whereas species-level entries matching the genus-level *Segatella* and Prevotellaceae NK3B31 group panels were omitted from the figure. Clostridiales bacterium CIEAF 012 increased from 0.0005 to 0.0057% (*p* = 0.0106; Figure 7E). *Akkermansia muciniphila* increased from 0.0044 to 0.1533% (*p* = 0.0110; Figure 7F). Bacterium NLAE-zl-P177 increased from 0.0496% in the pooled background to 0.4355% in RG (*p* = 0.0363; Figure 7G).

**Figure 7:**
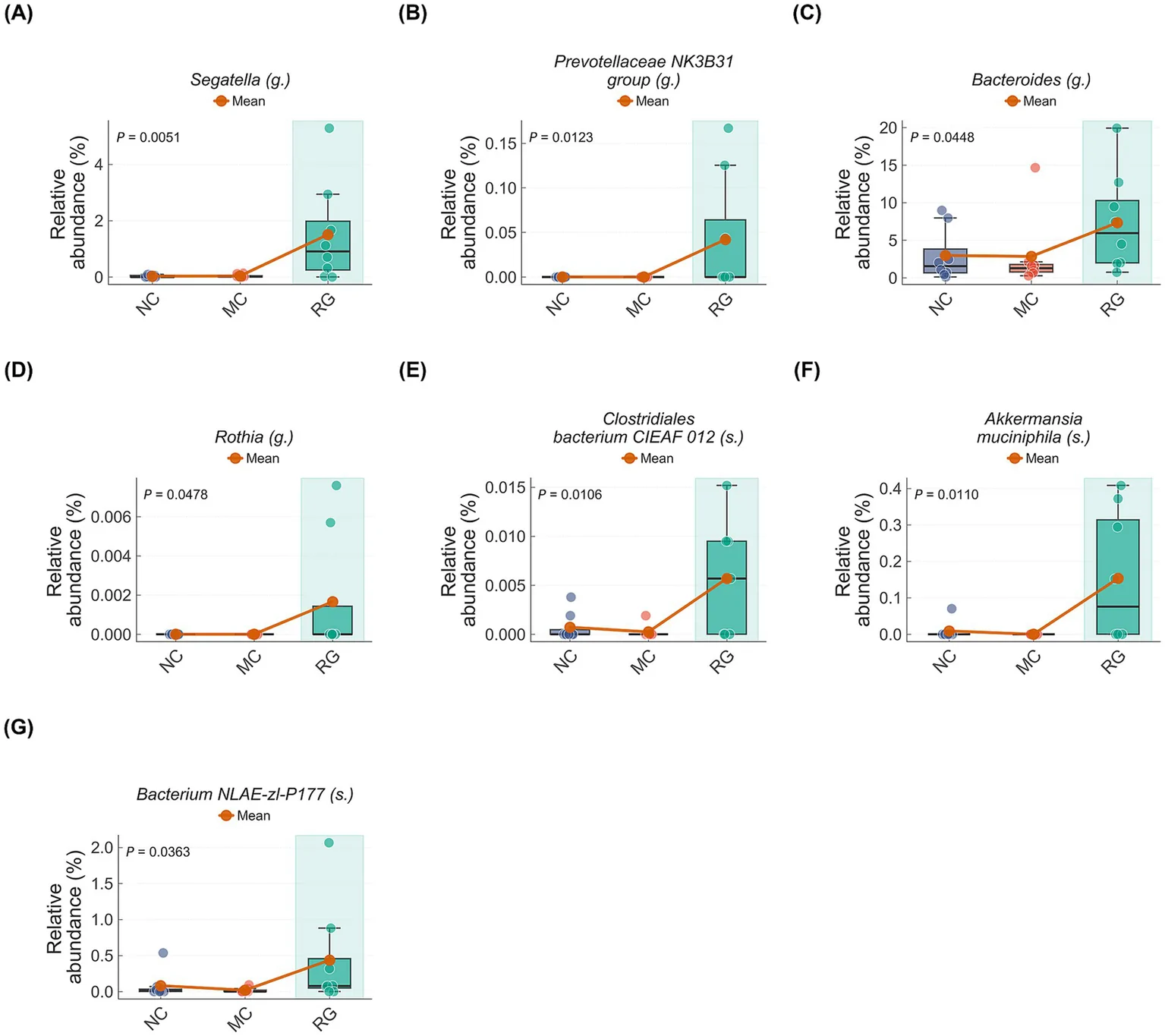
RG-enriched genus- and species-level taxa in the prevention experiment. Panels show the relative abundance across NC, MC, and RG as boxplots with jittered points; the red line indicates the group mean. **(A)** *Segatella*; **(B)** Prevotellaceae NK3B31 group; **(C)** *Bacteroides*; **(D)** *Rothia*; **(E)** Clostridiales bacterium CIEAF 012; **(F)** *Akkermansia muciniphila*; **(G)** bacterium NLAE-zl-P177. NC, normal control; MC, model control; RG, *Rothia mucilaginosa* group.

### 3.10 Colon structure and epithelial barrier markers

Representative H&E histology and ZO-1, Claudin-1, and MUC2 IHC are shown per group (Figures 8A–L). The colonic muscle-layer thickness did not differ between groups, showing only a non-significant downward trend from NC to RG (Figure 8M; pairwise Welch *t*-tests, all *p* > 0.05; NC vs. MC *p* = 0.0980, MC vs. RG *p* = 0.8268, and NC vs. RG *p* = 0.0867; source data are provided in Supplementary Table S2). H-scores for ZO-1, Claudin-1, and MUC2 likewise showed no significant pairwise differences between NC, MC, and RG (Figures 8N–P; all Welch *p* > 0.05; source data are provided in Supplementary Table S3).

**Figure 8:**
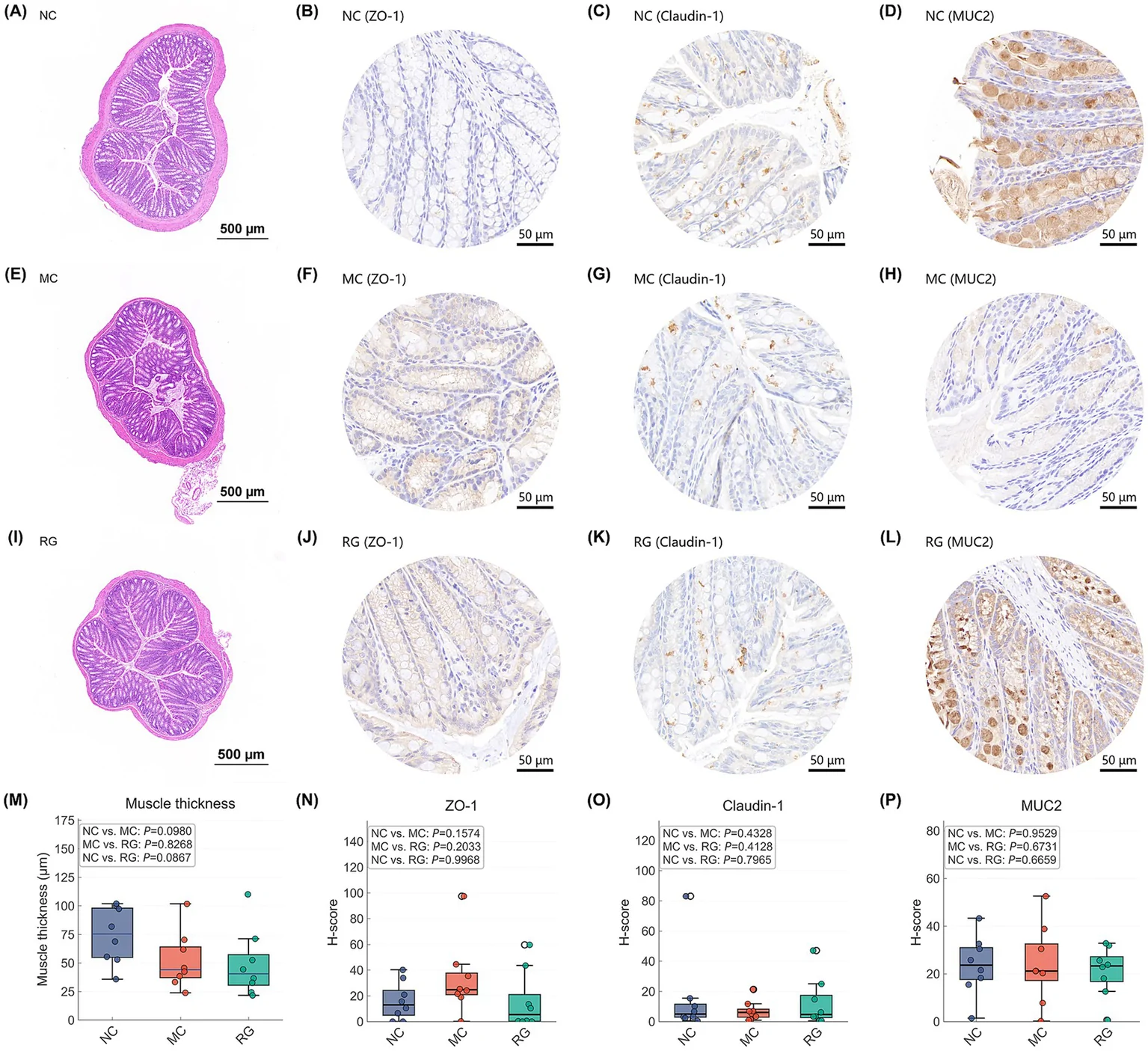
Colon histology and epithelial barrier integrity in NC, MC, and RG mice. **(A)** Distal colon H&E for NC. **(B)** ZO-1 immunohistochemistry for NC. **(C)** Claudin-1 immunohistochemistry for NC. **(D)** MUC2 immunohistochemistry for NC. **(E)** Distal colon H&E for MC. **(F)** ZO-1 immunohistochemistry for MC. **(G)** Claudin-1 immunohistochemistry for MC. **(H)** MUC2 immunohistochemistry for MC. **(I)** Distal colon H&E for RG. **(J)** ZO-1 immunohistochemistry for RG. **(K)** Claudin-1 immunohistochemistry for RG. **(L)** MUC2 immunohistochemistry for RG. **(M)** Muscle-layer thickness. **(N)** ZO-1 H-score. **(O)** Claudin-1 H-score. **(P)** MUC2 H-score. H&E, hematoxylin and eosin; IHC, immunohistochemistry; ZO-1, zonula occludens-1; MUC2, mucin 2; NC, normal control; MC, model control; RG, *Rothia mucilaginosa* group.

## 4 Discussion

Constipation is increasingly understood as a gut motility disorder embedded in host–microbiome crosstalk, as dysbiosis and microbial metabolites may influence intestinal movement through enteric and central neural, immune, secretory, hormonal, short-chain-fatty-acid, and 5-hydroxytryptamine-related pathways (Pan et al., 2022). In this context, the two-sample MR analysis in this study suggested nominally significant, genetically predicted protective associations of the genus *Rothia* and the species *R. mucilaginosa* with constipation; funnel plots were approximately symmetric and leave-one-out estimates remained stable, and the IVW and weighted-median estimates were concordant in the protective direction, but the genus-level MR-Egger slope was positive (*β* = +0.115) with a wide 95% CI, so the direction of effect was not fully consistent across the five methods; with only 6 SNPs for the genus and 4 SNPs for the species, the MR-Egger intercept tests had limited power to resolve directional horizontal pleiotropy. Conversely, genetically predicted Prevotellaceae and *Bacteroides plebeius* were associated with increased risk; the elevated *Bacteroides plebeius* is consistent with the compositional shifts reported in the constipated microbiota (Parthasarathy et al., 2016; Tian et al., 2020). Convergent sensitivity analyses argue against substantial horizontal pleiotropy for the *Rothia* signal; however, the reported *p* values are nominal and were not corrected for multiple comparisons across the 207 taxa; therefore, these MR findings are exploratory and warrant cautious interpretation and further validation. This protective effect may be biologically plausible because *R. mucilaginosa* is among genera persistently detected in the oral cavity and gut of healthy individuals, which is consistent with oral-to-gut translocation (Costa et al., 2024); as an oral commensal, this species secretes the metal-chelating siderophore enterobactin and attenuates pathogen-induced pro-inflammatory responses via NF-κB pathway inhibition in epithelial cultures and mouse models (Uranga et al., 2020; Rigauts et al., 2022).

In the *in vitro* metabolomic analysis, to distinguish genuine commercial *Rothia mucilaginosa* strain BNCC363031 from medium background, the supernatant and pellet upregulated sets were intersected, isolating 26 that were intracellularly accumulated and net secreted. As reported in the literature, the indole-3-pyruvate (IPyA) pathway is a major route of microbial tryptophan metabolism via 3-substituted indoles (Liu et al., 2022), and in this study the *in vitro* tryptophan metabolite profile of *R. mucilaginosa* is consistent with this pathway: ILA was identified as a net-produced candidate in the targeted tryptophan panel (supernatant versus medium) but was decreased in the untargeted pellet-versus-medium comparison; ILA is a reductive product of IPyA and has been reported to activate the aryl hydrocarbon receptor and Nrf2 signaling pathways, promoting tight junction protein expression and improving intestinal barrier function (Wang et al., 2023). Downstream along this branch, IPA was enriched in both the pellet-versus-medium and supernatant-versus-medium comparisons of the untargeted profiles, and has been reported to exert anti-inflammatory activity, including strengthening the epithelial barrier (Li et al., 2021) and alleviating colitis (Li et al., 2025); other reports show that ILA can be converted by other gut bacteria into IPA and indole-3-acetic acid (IAA) through cross-feeding, a conversion that requires an intact microbiota (Wang et al., 2024); however, IPA did not reach the differential threshold in the targeted tryptophan panel (supernatant versus medium) and showed the opposite direction to the untargeted result, possibly because untargeted peak-area signals are susceptible to matrix effects or co-eluting interferences; the exact cause warrants further investigation. Downstream along the IPyA pathway, indole-3-acetaldehyde (IAAld) was enriched in the pellet-versus-medium comparison, and a recent report identified IAAld as the predominant indole metabolite of *R. mucilaginosa* in isolated culture (Fan et al., 2026); IAAld can be converted to IAA, and IAA, in synergy with indole, has been shown to activate the pregnane X receptor (PXR) (Venkatesh et al., 2014), a xenobiotic sensor that regulates intestinal barrier function. In parallel, the targeted short-chain fatty acid panel (supernatant versus medium) identified acetic acid as a net-produced metabolite, consistent with complete-genome annotation of the key acetate-producing enzymes acetate kinase (ackA) and phosphotransacetylase (pta) in a prior *R. mucilaginosa* study (Jiang et al., 2026). Integrating the literature with the *in vitro* metabolomic results, whether these indole metabolites and acetic acid serve as mediators through which *R. mucilaginosa* exerts its effects, and whether they mediate its effects in the constipation model *in vivo*, remain to be determined.

A first-hour defecation burst after transfer to a fresh collection cage recurred stably across days 1, 7, and 14 without loperamide; interpreting this as a transient novel-environment stress response rather than a treatment effect is supported by rodent data showing that corticotropin-releasing factor (CRF)—a hypothalamic neuropeptide that coordinates the endocrine, autonomic, and behavioral stress responses—increases defecation during placement in a novel environment through CRF1 signaling, whereas selective CRF1 blockade reduced stress-induced defecation without altering basal colonic function in non-stressed animals (Williams et al., 1987; Tache et al., 2004; Forbes and Cox, 2014). Therefore, the modeling-phase comparisons were based on the summed last 3 h, favoring the post-acclimation window. Body weight remained statistically indistinguishable between NC, MC, and RG at every timepoint (all one-way ANOVA *p* > 0.05), indicating that loperamide-induced constipation in this protocol did not cause overt systemic wasting. In contrast, fecal water content—a recognized physicochemical index of the constipation phenotype—fell in MC and RG during modeling; therefore, a water-content deficit alongside unchanged body weight supports a functional, non-cachectic constipation model that frames the downstream defecation and barrier analyses. The day 15 last-3-h fecal pellet counts and wet mass totals, as well as the four-day (days 15/17/19/20) per-mouse means of these last-3-h outputs, indicate that loperamide suppressed defecation while preventive *R. mucilaginosa* suggested a partial rescue, with RG between NC and MC. Under two sensitivity analyses that excluded the reduced-dose day 19, the direction of the RG-versus-MC difference was retained across both the last-3-h window and a common post-dose 5–8 h window; however, significance was metric- and window-dependent: in the last-3-h window the pellet count remained significant (*p* = 0.040) while wet mass became a non-significant trend (*p* = 0.082), whereas in the fixed 5–8 h window the pellet count became a non-significant trend (*p* = 0.055) while wet mass remained significant (*p* = 0.025). Across both windows, however, the effect direction was consistent and the effect sizes were large (Cohen’s *d* 0.936–1.260), suggesting a rescue signal despite the wavering significance.

The OD600–CFU curve translated optical density into a delivered bacterial dose, and daily readings showed the gavaged *R. mucilaginosa* broth was administered below the 1 × 10^10^ CFU/mL target, with a back-calculated ~10^9^ CFU/mL (roughly 0.2–0.7 times target). Notably, the fecal 16S V4 signal corresponding to *R. mucilaginosa* on day 13 was group-restricted (Figure 5O), detected in only two out of eight RG mice at trace relative abundances and absent from NC and MC (0/8 each). *Rothia mucilaginosa* is a natural colonizer of the human upper gastrointestinal tract, being detected in the oral cavity (Zamakhchari et al., 2011) and in the proximal small intestine (Ou et al., 2009). In the animal experiment in this study, the fecal 16S V4 signal corresponding to *R. mucilaginosa* showed a low detection rate. This limited detection may reflect functional divergence between the commercial reference strain and gut-resident *R. mucilaginosa* (Desroches et al., 2018; Utter et al., 2020), together with the commercial strain’s limited capacity to adapt to the low-oxygen gut environment. It has been reported that live and dead microbial cells can confer benefits via secreted metabolites and structural components acting on host pattern-recognition receptors without stable engraftment (Adams, 2010). The benefit observed in this animal experiment after *R. mucilaginosa* intervention may therefore stem from transient exposure, or from the commensals and metabolites it transiently favors, rather than from persistent colonization. The *in vitro* candidate metabolites identified from *R mucilaginosa* cultures (acetic acid, IAAld, ILA) may act as postbiotic effectors during their transient passage through the distal gut, and their *in vivo* activity in the constipation model warrants further verification. In parallel, the resident microbiota was also reshaped: in the RG group, the more robust enrichments included carbohydrate-fermenting commensals *Bacteroides* and *Segatella*, both encoding polysaccharide utilization loci for dietary plant glycans such as mixed-linkage β-glucans, xylans, and pectins (Panwar et al., 2025), and the mucin-associated *Akkermansia muciniphila* (Derrien et al., 2004; Koropatkin et al., 2012). *Akkermansia muciniphila* is a mucus-layer-associated gut commensal adapted to host glycan utilization, placing it at the interface between the mucosal niche and community metabolism (Koropatkin et al., 2012). Its enrichment after RG intervention (Figure 7F) echoes the earlier therapeutic *Rothia mucilaginosa* study, which also reported increased *Akkermansia muciniphila* among enriched beneficial bacteria (Jiang et al., 2026). The recurrence of this signal in the present prevention-oriented setting suggests that *Akkermansia muciniphila* may be a reproducible microbiota feature associated with *R. mucilaginosa* exposure.

In the *in vivo* endpoint analyses, colonic muscle-layer thickness did not differ significantly between NC, MC, and RG, suggesting no overt muscularis remodeling in this model. ZO-1, Claudin-1, and MUC2 are commonly used to assess intestinal barrier integrity (Capaldo et al., 2017); although IPyA pathway metabolites have been reported to enhance intestinal epithelial barrier markers including claudin-1, ZO-1, and MUC2/MUC4 (Li et al., 2021; Wang et al., 2023), and the *in vitro* metabolomics in this study suggests that *R. mucilaginosa* may harbor an active IPyA pathway, terminal IHC detected no corresponding changes in ZO-1, Claudin-1, or MUC2 H-scores. The modest effect observed in this exploratory test of the commercial *R. mucilaginosa* strain therefore cannot, for now, be attributed to detectable barrier-marker changes.

This study has several limitations. The reported *p* values were nominal and uncorrected for multiple testing across 207 taxa; therefore, the *Rothia* signal is hypothesis-generating rather than a confirmed causal effect. The *in vivo* experiments used a small, single-sex cohort (male C57BL/6 mice, *n* = 8 per group) and a single loperamide-induced constipation model, which may limit generalizability to female animals, other genetic backgrounds, and human chronic or idiopathic constipation. The day 19 time point is limited in interpretability because the reduced loperamide dose (10 mg/kg) used on that day did not maintain the constipation phenotype. Moreover, day 20 used an earlier gavage time, placing its collection windows in different circadian periods, so the mean across modeling days cannot fully exclude circadian rhythm confounding. In addition, the strain used here (BNCC363031) is a commercial reference strain rather than an isolate derived from the human gut, and was detected in only a few mice at trace abundance after gavage; whether it can functionally mimic the *R. mucilaginosa* strain identified by MR remains unknown; the animal experiment should therefore be regarded as exploratory testing of a commercial *R. mucilaginosa* strain, and future work using isolates derived from the gut and characterized at the strain level is needed to conduct experiments that provide validation concordant with the MR signal; whether facilitated colonization, such as antibiotic pretreatment to deplete the resident microbiota prior to gavage, would enhance the preservation of fecal output by this commercial strain also remains to be determined in future studies.

## 5 Conclusion

Two-sample MR suggested nominally significant, genetically predicted protective associations of the genus *Rothia* and the species *R. mucilaginosa* with constipation. As exploratory testing of a commercial *R. mucilaginosa* strain, intragastric gavage in a preventive loperamide model partially preserved fecal output in the early period after gavage ended; however, epithelial barrier markers at terminal sacrifice were unaltered, and stable colonization was not demonstrated by the available data.

## Data Availability

The data generated in this study are publicly available in the MetaboLights repository under study accession MTBLS14792 (https://www.ebi.ac.uk/metabolights/MTBLS14792) for untargeted LC-MS metabolomics data from in vitro cultured *Rothia mucilaginosa* supernatant and pellet samples, and under study accession MTBLS15044 (https://www.ebi.ac.uk/metabolights/MTBLS15044) for targeted LC-MS/MS metabolomics data (tryptophan and short-chain fatty acid panels) from in vitro cultured *Rothia mucilaginosa* supernatant samples; and in the NCBI Sequence Read Archive (SRA) under BioProject accession PRJNA1478980 (https://www.ncbi.nlm.nih.gov/sra/PRJNA1478980) for 16S rRNA gene amplicon sequencing data from the loperamide-induced constipation mouse model with preventive *Rothia mucilaginosa* intervention. Publicly available GWAS summary statistics used in this study were obtained from the Dutch Microbiome Project gut metagenome GWAS (GCST90027446-GCST90027857) and the European constipation GWAS GCST90018829, as described in the Methods. Mouse colon H&E and immunohistochemistry (IHC) whole-slide images are publicly available from Figshare at: https://doi.org/10.6084/m9.figshare.32955605.

## Supplementary material

SUPPLEMENTARY TABLE S1 Mouse-level source data for daily fecal pellet count, wet fecal mass, and fecal water content corresponding to Figures 3–6.

SUPPLEMENTARY TABLE S2 Mouse-level colon muscle-layer thickness source data corresponding to Figure 8M.

SUPPLEMENTARY TABLE S3 Mouse-level ZO-1, Claudin-1, and MUC2 H-score source data corresponding to Figure 8N–P.

SUPPLEMENTARY TABLE S4 Complete two-sample Mendelian randomization screening results for gut taxa with multiple-testing-adjusted *p* values.
